# Embryo-Engineered Nonhuman Primate Models: Progress and Gap to Translational Medicine

**DOI:** 10.34133/2021/9898769

**Published:** 2021-08-21

**Authors:** Mei Huang, Jiao Yang, Peng Li, Yongchang Chen

**Affiliations:** ^1^Yunnan Key Laboratory of Primate Biomedical Research, Institute of Primate Translational Medicine, Kunming University of Science and Technology, Kunming 650500, China; ^2^State Key Laboratory of Primate Biomedical Research, Kunming 650500, China

## Abstract

Animal models of human diseases are vital in better understanding the mechanism of pathogenesis and essential for evaluating and validating potential therapeutic interventions. As close relatives of humans, nonhuman primates (NHPs) play an increasingly indispensable role in advancing translational medicine research. In this review, we summarized the progress of NHP models generated by embryo engineering, analyzed their unique advantages in mimicking clinical patients, and discussed the remaining gap between basic research of NHP models to translational medicine.

## 1. Introduction

Animal models of human diseases are the most important foundation for revealing the pathogenesis of diseases and exploring more effective therapeutic strategies. The available approaches to generate animal models of human disease generally include spontaneous animal models [1, 2], induced animal models [3–7], and embryo engineering animal models. Animal models with spontaneous mutation are formed in nature without human intervention. Although they could reflect clinical and pathological characteristics of the disease, they have shortcomings of difficult sources and few types, which greatly limit their applications. There are various methods used to induce animal models, including physical [3], biological [4–6], chemical [7], and/or other pathogenic factors. Among them, chemical and physical methods can generate animal models in a short time but they are difficult to reveal the disease process. By contrast, disease animal models constructed by embryo engineering can not only explore the treatment of diseases but also monitor the dynamic disease progression from an early stage.

**Figure 1:**
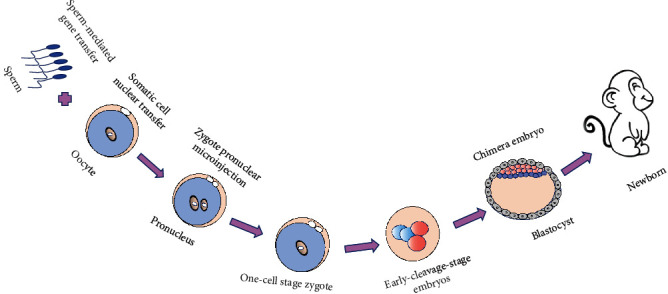
Schematic diagram of genetically modified animal models using embryo engineering.

Due to the development of assisted reproductive technologies (ARTs), such as *in vitro* fertilization (IVF) [8], intracytoplasmic sperm injection (ICSI) [9], and embryo culture technology *in vitro*, mammalian embryo development has been gradually explored. The development of mammalian embryos includes preimplantation and postimplantation development [10, 11]. After the oocyte is fertilized in the ampulla of the fallopian tube, the fertilized egg, known as the zygote, undergoes rapid mitotic division, known as cleavage. A cluster of 16 identical blastomeres is called morula. The blastomeres continue to divide to form blastocyst with an inner cell mass and trophectoderm. After the zona pellucida disappears, the blastocyst implants into the uterus to develop into an individual [12, 13]. During the preimplantation stage, there is a time window for engineering embryos. Several methods can be used during the window, including the sperm carrier method, which modifies the sperm [14], somatic cell nuclear transfer (SCNT), which works at the stage of mature oocytes [15, 16], microinjection of the viral vector or gene editing system, which works at the multiple stages [17–20], or embryonic stem cell (ESC) method to get the chimera [21, 22] (Figure 1).

Animal models constructed by embryo engineering have played an important role in understanding gene functions and molecular mechanisms of diseases. However, many small animals are different from humans in terms of genetics and evolution. Small animal models of the disease have shortcomings in simulating disease processes and pathological phenotypes, which cannot be ignored. Due to the high similarities between nonhuman primates (NHPs) and human beings, NHP models have been recognized as an indispensable bridge from basic research to clinical transformation. In this review, we systematically reviewed the research progress of generating NHP disease models using embryo engineering and discussed the advantages and disadvantages of the NHP models. Finally, solutions to NHP model construction problems and potential applications of NHP models in translational medicine are prospected.

## 2. Development of the NHP Models

Although there are numerous technical and ethical limitations, many NHP disease models were constructed after unremitting exploration, even produced offspring models with a uniform genetic background. The first transgenic NHP model was generated 20 years ago [17]. Seven years later, the first gene-edited NHP disease model was constructed [23]. In general, more than 20 types of different gene-edited NHP models have been successfully generated in the past 20 years (Table 1). Here, we reviewed the existing gene-edited NHP models and major achievements from five aspects: viral vector-mediated transgenic NHP models, precise knockout genetically modified NHP models, precise knockin genetically modified NHP models, precise point mutation NHP models, and somatic cell nuclear transfer NHP models, to gain experience and develop translational medicine.

### 2.1. Viral Vector-Mediated Transgenic NHP Models

This method uses viral vectors to integrate related genes into the target cells. Chan and his colleagues successfully produced the first genetically modified monkey in 2001 [17]. They injected the pseudotyped replication-defective retroviral vector into the perivitelline space of 224 mature rhesus oocytes. Oocytes were fertilized by intracytoplasmic sperm injection (ICSI) and 40 embryos were transferred to 20 surrogates. One out of three newborns were detected to contain the *GFP* gene integration. Although the transgenic mice accelerated the advancement of biomedical sciences to a great extent, many differences between humans and rodents cannot be ignored. The advent of genetically modified monkeys that are more similar to humans was undoubtedly a significant breakthrough.

Based on the same technology, the first transgenic monkey model of Huntington's disease (HD) was created in 2008 [23]. Yang et al. injected a high-concentration lentivirus carrying 84 CAG trinucleotide repeats of the human huntingtin *(HTT)* gene (*HTT-84Q*) into the perivitelline space of mature rhesus oocytes and successfully constructed five HD rhesus monkeys. Generally, healthy individuals contain up to 36 CAG repeats, whereas the disease occurs when the number of CAG repeats increase to more than 40. CAG repeat expansion translates into polyglutamine which further forms the polyQ aggregates, a master pathological feature of HD patients [24]. By studying those five HD monkey models, Yang et al. found that the levels of mutant HTT in the tissues seem to be associated with the severity of illness. This supported the theory that N-terminal mutant HTT fragments are pathogenic. The successful construction of gene-edited disease monkey models has opened up a new avenue for studying disease mechanisms and treatment strategies. It supplements the deficiencies of other animal models, especially mouse models.

Compared with Old World monkeys, such as rhesus monkeys and cynomolgus monkeys, the New World common marmosets (*C. jacchus*) have shorter reproductive cycles and smaller sizes. Therefore, using marmosets as animal models to study complex human diseases is faster and more feasible. In 2009, Sasaki et al. obtained different embryos through natural intercourse and IVF. By injecting lentiviral vectors carrying the enhanced green fluorescent protein (*EGFP*) gene into the perivitelline space or blastocyst cavity of early embryos, they successfully obtained five transgenic marmosets expressing the *EGFP* gene [18]. This is the first successfully constructed transgenic marmoset model, providing an efficient and practical animal model for the biomedical field. Moreover, they collected semen samples from the first-generation (F_0_) monkey model to obtain an offspring that carried the *EGFP* gene and proved the lineage inheritance of transgenic monkeys. However, the expression organization and strength of the five first-generation transgenic marmosets were not the same and the copy of *EGFP* and the integration site were also different, suggesting a random integration and uncontrollable expression strength. This is the common disadvantage of constructing transgenic monkey models with viral vectors. Following *GFP* transgenic rhesus monkeys and common marmosets, *GFP* transgenic cynomolgus monkey has been generated by Seita et al. [25]. They compared and analyzed the effects of lentivirus injection before and after fertilization and found that cynomolgus monkeys with lentivirus injection before fertilization can express *GFP* all over the body, indicating that the earlier the virus injection, the easier to obtain a homozygous animal model. However, injecting virus before fertilization can increase the rate of miscarriage, raising the question that the efficiency of virus integration and the development rate of embryos need to be balanced.

In 2010, the first genetically modified monkey in China was created by infecting embryos with simian immunodeficiency virus (SIV) in the early cleavage stage [26]. Two living infant monkeys expressed *EGFP* stably. Both *in vivo* and *in vitro* experiments showed that SIV infection had no significant effect on the development rate of embryos compared to the noninfected negative control group. The construction efficiency of transgenic monkeys has been significantly improved by improving and developing ARTs and using an SIV-based vector to infect early cleavage-stage embryos. However, flow cytometry showed that less than 30% of the cells from the transgenic baby monkeys expressed *EGFP*, suggesting that only part of the early embryonic cells was infected by the virus, resulting in the transgenic rhesus monkey chimeric mutants.

Besides HD, more transgenic NHP disease models have been reported. Parkinson's disease (PD) models were established in rhesus monkeys by Niu et al. in 2015 [27]. Interestingly, in the lentiviral vector, mutant *α-syn* (*A53T*) and *ECFP* are linked via F2A (which could self-cleave in cells after transcription). In this way, the length of the integrated fragment is reduced due to one vector expressing two proteins. It is also convenient to judge whether the mutant gene is integrated successfully by detecting the expression of ECFP protein. It is worth mentioning that the transgenic PD monkey models showed similar nonmotor symptoms to humans, which provided a suitable animal model to monitor early nonmotor symptoms of PD. Nonmotor symptoms have a significant impact on patients. However, the clinical research is hindered by lacking data in early stages of PD and difficulties in follow-up of patients. Therefore, it is extremely important and necessary to identify biomarkers and treatment strategies based on established PD monkey models. The autism-like syndrome in cynomolgus monkeys was reported by Liu et al. in 2016 [28]. These monkeys carry *methyl-CpG binding protein 2* (*MECP2*) gene duplication. They injected lentivirus carrying the hSynapsin-HA-hMECP2-2a-GFP cassette into the perivitelline space of mature oocytes. The F_0_ monkey models expressed the human *MECP2* gene in their brain and exhibited autism-like behaviors. The F_1_ monkeys carried the human *MECP2* gene and displayed reduced social activities, proving the success of germline transmission of the transgene. Recently, Cai et al. discovered that *MECP2* coexpressed genes significantly enriched in GABA-related signaling pathways, which could reduce beta synchronization within fronto-parieto-occipital and clarify abnormal locomotive behaviors in *MECP2* duplication syndrome individuals [29]. The findings based on this model imply the feasibility and reliability of using genetically engineered NHPs to study psychiatric diseases.

The evolution and development of the human brain have always been a research hotspot. Nonhuman primates are ideal experimental animals for studying the evolution of the human brain. After birth, the expression level of human *MCPH1* is high during brain development but the expression level of *MCPH1* in nonhuman primates such as monkeys is relatively low. This is perhaps due to the unique structure and function of human *MCPH1* in the human brain. In order to explore the function of this gene, Shi et al. used lentivirus transfection to overexpress human *MCPH1* in rhesus monkeys [30]. The results showed that the transgenic monkeys exhibited human-like brain developmental neoteny and exhibited enhanced short-term memory, but not enlarged brain size as expected. These suggested that a single genetic change cannot simulate the complex evolution of the human brain.

In general, using viral vectors to integrate exogenous genes into the embryonic genome is the earliest technology to generate transgenic monkeys. Initially, the exogenous gene is *GFP* or *EGFP* as a reporter; later, some pathogenic genes were integrated to generate disease models. Transgenic monkeys have made great contributions to disease research; however, it is only limited to the disease that could be induced by exogenous gene expression. Moreover, viral vector-mediated gene transfer has other fatal limitations, such as the gene size of insertion, uncontrollable expression strength, and random insertion [31]. In order to overcome these shortcomings, the second-generation gene editing technology has been developed.

### 2.2. Precise Knockout Genetically Modified NHP Models

The three most popular methods of second-generation gene editing technologies are zinc finger nucleases (ZFNs), transcription activator-like effector nucleases (TALENs), and clustered regularly interspaced short palindromic repeat/Cas9 (CRISPR/Cas9) [31, 32]. Here, we reviewed the published targeted gene knockout monkey models generated by these three methods.

Liu et al. successfully constructed the first TALEN-mediated *methyl-CpG binding protein2* (*MECP2*) knockout rhesus and cynomolgus monkeys. According to PCR and gene sequencing analysis, no off-target or plasmid integration was found on the genome [33]. *MECP2* is an X-linked gene, which is extremely essential to the growth and development of humans and monkeys. Duplication of *MECP2* gene will cause *MECP2* duplication syndrome, showing similar symptoms to autism, whereas *MECP2* gene loss-of-function mutations will cause Rett syndrome (RTT), a severe neurodevelopmental disorder [28, 33]. The monkey model of RTT syndrome showed similar physiological, behavioral, and brain structural abnormalities to human RTT symptoms [34]. Therefore, it is a great opportunity to analyze the pathogenic mechanism and treatment methods of RTT through an in-depth exploration of monkey models. Ke et al. used TALEN to knockout biallelic *MCPH1* and generated a cynomolgus monkey model of autosomal recessive primary microcephaly (MCPH), a genetically neurodevelopmental disorder [19]. The MCPH1 protein can inhibit the expression of telomere reverse transcriptase. If the *MCPH1* gene mutation leads to a decrease in the MCPH1 protein level, the patients will experience a significant reduction in brain size and height. Some patients will have decreased intelligence and other neurological diseases. The main feature in patients with MCPH at the cellular level is premature chromosome condensation (PCC), which also appeared in the monkey MCPH model. Sato et al. used ZFN and TALEN technologies to target the *IL2RG* gene and constructed five and four X-linked severe combined immunodeficiency (X-SCID) marmoset models, respectively [35]. This is the first batch of monkey models constructed by ZFNs. By injecting mRNA into embryos at the prokaryotic stage and analyzing it after culturing for seven days, TALEN was found to have a higher mutation rate than ZFNs. This may be one of the reasons why TALENs are used more often than ZFNs, although both of them are not commonly used in gene editing.

Due to the difficulties and time-consuming operations of ZFNs and TALENs, more and more genetically modified monkey models are now constructed by CRISPR/Cas9. CRISPR/Cas9 is simpler to operate, more versatile, and more efficient. In 2014, the same year that TALEN was first used to construct monkey models, the first monkey model that used CRISPR/Cas9 was also constructed successfully. Niu and colleagues injected Cas9 mRNA and gRNAs targeting *Nr0b1*, *Ppar-g*, and *Rag1* into one-cell-stage embryos [36]. *Ppar-g* and *Rag1* knockout cynomolgus monkey models were born when the article was published. However, no *Nr0b1* gene mutation was detected in the newborn monkeys. One year later, a total of 3 surviving cynomolgus monkeys were born by the remaining 8 pregnant female monkeys but only one of the newborn monkeys was detected with the *Rag1* mutation. Moreover, mutations have been detected in the somatic and germ cells of aborted fetuses, suggesting that gene mutations caused by the CRISPR/Cas9 technology can also be transmitted through the germ line [37]. In 2015, three knockout monkey models based on CRISPR/Cas9 were published, including the *P53* knockout cynomolgus monkey model [20], Duchenne muscular dystrophy (DMD) rhesus monkey model [38], and X-linked adrenal hypoplasia congenita-hypogonadotropic hypogonadism (AHC-HH) cynomolgus monkey model [39]. Here, we focused on the research progress of DMD in the NHP models. DMD is an X-linked recessive genetic disease. Patients are mostly male and the incidence is about 1/3500 to 1/5000 [40–42]. The main symptom of DMD is the deterioration of muscle function. The patient died from respiratory failure or heart failure around the age of 30s. In recent years, due to the use of ventilators to treat DMD patients with respiratory decline, the death of DMD patients has declined, which means that the death ratio of heart failure has gradually increased [43]. In 2015, a rhesus monkey model of DMD was generated [38] with no off-target occurrence [44]. Through the exploration and study of this model, it is very likely that new treatments for DMD could be developed. In 2017, the first cynomolgus monkey model of autism spectrum disorders (ASD) caused by *SHANK3* gene mutation was generated by Zhao et al. [45]. The strategy of mutating the *SHANK3* gene is to target the 6th and 12th exons of the *SHANK3* gene, resulting in large fragment deletion of the *SHANK3* gene. They implanted 116 embryos into 37 surrogate mothers, and only three female monkeys were pregnant. The pregnancy rate was extremely low, which is only 8.1%. The exact reason for the low pregnancy rate is unknown, but it suggests that the expression of the *SHANK3* gene is very important for the early development of primates. In 2018, Zhang et al. constructed a cynomolgus monkey model with biallelic *SIRT6* mutations, which provides a suitable model for studying human perinatal lethality syndrome [46]. *SIRT6* gene deletion delays neuronal differentiation through transcriptional activation of long non-coding RNA H19 (a developmental inhibitor). The cynomolgus monkey model with *SIRT6* mutation exhibits delayed embryonic development and died soon after birth. In 2019, Zhou et al. targeted exon 21 of the *SHANK3* gene to construct five cynomolgus monkey models with ASD and Phelan–McDermid syndrome [47]. The F_1_ generation monkey model was generated using F_0_ generation germ cells, which made up for the shortcomings of an insufficient number of *SHANK3* mutant monkey models. It is worth noting that there is a homozygous model for the mutant allele in the F_0_ generation, suggesting that the development of the CRISPR/Cas9 technology will eventually increase the mutation rate and mutation homozygous rate of the gene. Qiu et al. constructed five *BMAL1* gene knockout circadian disruption cynomolgus monkey models, providing a suitable animal model for studying biological rhythm disorder [48]. Tsukiyama and colleagues constructed a monkey model of autosomal dominant polycystic kidney disease (ADPKD), an autosomal dominant genetic disease with a high incidence [49]. Yang et al. constructed a PD monkey model with large fragment deletion of the *PINK1* gene. The monkey model showed similar symptoms of neuronal loss in humans, which has not been observed in mice or other models [50]. It is known that gene therapy may cure *β*-thalassemia; however, there is no suitable animal models for evaluating the safety and efficacy of this therapeutic strategies *in vivo*. Huang et al. constructed a *HBB*-deficient *M. fascicularis* monkey model that could be used to study the mechanism of *β*-thalassemia and the long-term safety and efficacy of gene editing therapies targeting *HBB* [51]. Since the mouse models of ADPKD, PD, and *β*-thalassemia exhibit large differences to humans in physiology, monkey models of these diseases made up for the shortcomings of the mouse models, providing an ideal way to study disease pathogenesis and discover therapy strategies.

In summary, with the development and improvement of the second-generation gene editing technology, more and more genetically precise knockout NHP models have been constructed. These models provide a suitable platform for studying the mechanism of complex diseases and the exploration of treatment methods. Compared with retroviruses, gene knockout based on the second-generation gene editing technology has a significant advantage of controllable edit sites. But there are still many shortcomings in the genetically precise knockout technology. For example, CRISPR/Cas9 has the disadvantages of low gene knockout efficiency, mosaicism, restriction of PAM sites, and off-target effects.

### 2.3. Precise Knockin of Genetically Modified NHP Models

The second-generation gene editing technology can activate DNA damage repair mechanisms in cells by targeting double-strand breaks (DSBs) [52]. DNA damage repair mechanisms include nonhomologous end-joining (NHEJ) and homology-directed repair (HDR) [53]. NHEJ is the primary and efficient DNA damage repair mechanism. It will cause indel (including insertion or/and deletion) mutations while repairing DNA damage. HDR repair occurs in the presence of a donor template. Through the specific design of the donor template, specific gene knockout or knockin could be achieved. The existence of the template makes the gene editing more accurate, which can achieve the true “precision gene editing.” However, the repair efficiency of HDR is very low. Kumita et al. verified the efficiency of specific gene knockin and knockout at the embryo level [54]. They found that the knockout efficiency of *c-kit* and *Shank3* can reach more than 70% and even 100% editing efficiency. But *c-kit* knockin efficiency is only about 30% under optimal conditions.

In spite of technical difficulties, in recent years, accurate gene knockin animal models have been successfully constructed. In 2017, based on the principle of HMEJ and CRISPR/Cas9 technology, Yao et al. successfully conducted the precise *mCherry* gene knockin in cells, mouse embryos, monkey embryos, and mice, providing a new way to generate animal models [55]. In 2018, Cui *et al.* constructed the first *Oct4-hrGFP* precision knockin monkey models, achieving a major breakthrough in the gene editing field [56]. But the NHEJ repair is detected, and the HDR repair efficiency needs to be further improved. HDR repair is a promising gene repair mechanism, because it can achieve multiple types of gene mutations and does not have the uncertainty like NHEJ repair. The research and development of HDR repair will bring tremendous progress in the field of life sciences.

### 2.4. NHP Models via Precise Point Mutation

Genetic diseases are caused by multiple types of gene mutations, including point mutations, deletions, duplications, increase or decrease in copy numbers, indels, and insertions. Among them, the top three pathogenic gene mutations are point mutation, deletion, and duplication. It is worth noting that point mutations account for more than 50% of all pathogenic mutation types and it is mainly based on the change of AT to GC base pair (about 47.5% of point mutations) [57]. Since the CRISPR/Cas9 system often causes nonideal mutations such as indels and large fragment deletions, it seems not efficient and reasonable to construct animal models with single point mutations by CRISPR/Cas9. To address these difficulties, base editors (BEs) came into being. The BE system requires three elements: a Cas9 nickase fused to a nucleobase deaminase enzyme, a gRNA targeting Cas9 to a specific locus, and DNA glycosylase inhibitor. It includes cytosine base editors (CBE) (C to T mutation) and adenine base editors (ABE) (A to G mutation), which could cause precise point mutations in the target window without completely breaking the DNA double strand [58, 59]. This determines that BEs could introduce single point mutations more efficiently and more beneficial for modeling-related diseases. In 2020, Wang et al. injected BE mRNA and sgRNA targeting the *LMNA* gene into monkey zygotes to construct the Hutchinson-Gilford progeria syndrome (HGPS) monkey model, which is caused by single point mutation (1824 *C* > *T*) [60]. Mutations in *LMNA* can cause the accumulation of a toxic-truncated protein called progerin, causing changes in the structure and function of the nuclear membrane and ultimately leading to premature aging of children. At present, the treatment of this disease has been explored using CRISPR/Cas9 at the cellular level and in the HGPS mouse model. The results showed that gene editing therapy alleviated the phenotypes in cells and symptoms in mice models, manifested by the moderated nuclear phenotype and the prolonged life span of the mouse model. However, because of the organ targeting limitation of the adeno-associated virus (AAV), the immunohistochemical results showed that the nuclear structure of liver, heart, and skeletal muscle cells in mice improved significantly but there was no significant improvement in the lung, kidney, and aorta [61]. Zhou et al. verified the efficiency of the BE system at the embryonic level in mice and macaques, providing an effective reference for the later establishment of T158M mutant RTT animal models [62]. Zhang et al. coinjected SpCas9-based ABE mRNAs, SaCas9-based SaKKH-BE3 mRNAs, and their corresponding sgRNAs into monkey embryos and found that CBE and ABE can function in the same cell, suggesting an effective treatment strategy for polygenic diseases [63]. The NHP models will further improve our understanding of diseases and help us to comprehensively and accurately evaluate the safety and effectiveness of the drug before the clinic.

### 2.5. NHP Models via Somatic Cell Nuclear Transfer (SCNT)

Because germ cells are difficult to culture and differentiate *in vitro*, they are mostly obtained *in vivo*. However, the number of germ cells is very limited, which greatly affects the development of NHP models. Somatic cell nuclear transfer (SCNT) enables CRISPR/Cas9 to edit somatic cells; therefore, the edited cells can be cultured *in vitro* to expand the number of cells. Part of the cells is used for genetic sequencing. If the sequencing is qualified, the remaining cells are transferred to the enucleated oocytes and finally transplanted to the surrogate mother to produce genetically uniform monkeys.

After Dolly the cloned sheep was produced in 1996 [15], many somatic cell-cloned mammals have been reported. However, as the closest evolutionary relative to humans, the somatic cell-cloned NHPs have not been successfully constructed. It is the problem that the whole world is looking for a breakthrough. In 2018, Chinese scientist Liu et al. used histone demethylase and deacetylase inhibitors to increase the embryonic development rate of somatic cell clones and successfully constructed two SCNT cynomolgus monkey models [16]. In 2019, they isolated fibroblasts from a CRISPR/Cas9-mediated *BMAL1* knockout monkey model [48] and successfully cloned five homozygous macaque monkeys with *BMAL1* mutation-induced biological rhythm disorder without mosaicism [64]. The success of NHP SCNT provides a new and efficient way to construct NHP models with uniform genetic backgrounds.

Now, we can obtain gene-specific knockin, knockout, or point mutations NHP models through embryo engineering. However, gene editing technologies and the construction of NHP models still have many imperfections.

## 3. Challenges and Opportunities of Existing Animal Models

### 3.1. Shortcomings of Existing Animal Models

Rodents are the most widely used experimental animal models (more than 90%) [65–73]. Although the mouse model is low cost and easy to operate, it has limitations in revealing human biology. The biological system of mice, especially the immune system, is not completely consistent with the human immune system. This is characterized by many differences in the interaction of innate immune molecules. For example, mice can express immune molecules TLR11, TLR12, and TLR13 when lacking the functional immune molecule TLR10. However, humans cannot express those three immune molecules if lacking functional TLR10. Many experimental studies have been carried out in immunodeficient mice; therefore, it is difficult for the mouse model to truly simulate the immune response process of the human immune system, which reduces the reliability of prediction in clinical applications. Many human pathogenic factors and drugs are species specific. Some pathogens are only targeted primates, not mice. All of these problems directly affect the smooth transformation from biomedicine to clinical practice. In recent years, more and more researchers tend to use large animal models such as pig models. With more than 80% similarities in analysis parameters, domestic pigs are closely related to humans in terms of the immune system. Therefore, pigs can be used as a powerful animal model to study immune diseases [74, 75]. Pigs are also potential organ donors for humans. NHPs transplanted with pig organs can survive for months or even years [76]. This renewed the interest in its potential to ease organ shortages. However, widespread application of pig organ transplantation is limited by immunosuppressive complications, chronic rejection. In addition, porcine embryonic stem cell resources have not been widely developed and the culture system and differentiation system are not mature enough. These further limited the application of pigs as animal models.

### 3.2. Advantages and Challenges of NHP Models

NHPs are very similar to humans in the central nervous system, immune system, and cardiovascular system [77], NHPs played a vital role in scientific research in recent years. The similarity between NHPs and the human genome is as high as 98%, which can be used to extensively study human-related genetic diseases. NHPs have a sulcus structure and a well-developed prefrontal lobe, which are significantly important for study and memory. It is an excellent animal model for the research of central nervous system-related diseases. Currently, NHP models for Parkinson's disease [27, 50], microcephaly [19], autism [28, 45], and other related diseases have been established and breakthroughs in those areas are expected. Meanwhile, NHPs can serve as important models for developing and evaluating effective treatment strategies. Because of those advantages, NHP models have become more and more vital in recent years [78–82].

**Figure 2:**
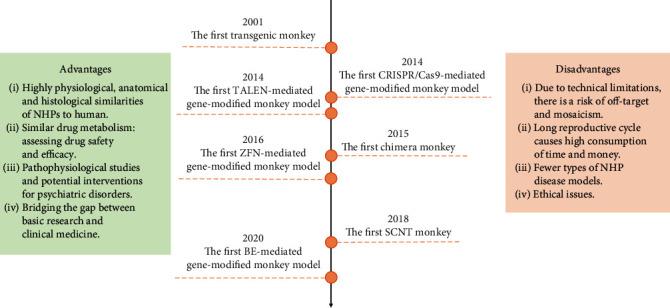
Major breakthroughs of the NHP models and their advantages and disadvantages.

However, there are also many challenges in the application of NHP models, such as the reproductive cycle is longer than other experimental animals and the genetic manipulation is difficult. A study in crab-eating monkey embryos showed that the targeting rate of CRISPR/Cas9-based gene editing is not precise enough and the single-nucleotide polymorphism (SNP) effect also needs to be considered [36]. Moreover, in a study of somatic cell-cloned monkeys, the survival rate after nuclear transplantation with cumulus cell or monkey fetal fibroblasts is very low [16], which brings great difficulties to the follow-up work. However, as long as the NHP models are constructed, the characteristics of NHPs are incomparable by any other animals. They are closer to the real situation of human beings and also have higher persuasion and credibility. In general, compared with other animal models, NHPs have unique advantages, but meanwhile, they also face many challenges (Figure 2).

### 3.3. Quantity and Cost of NHP Models

In the past few years, the use of animal models has gradually increased. Mice are the dominant animal models because of their low price and ease of operation. Although nonhuman primates are small in number and difficult to operate, they still have an irreplaceable position in the research of human genetic diseases and neurological diseases. At present, more than 170000 NHPs are used in biomedical experiments worldwide each year. Among them, the vast majority of NHPs (about 70000) were used by the United States [83]. Europe and Japan are also in the leading position. Compared with other animal models, even with other large animal models, the number of successfully constructed NHP models is very low. The main reasons include the low mutation rate of embryos and the low survival rate of birth. In terms of the cost of animal models, as the experimental value of monkeys becomes more and more prominent, the cost of buying monkeys is rising, from US $2000 to more than US $10000, plus daily feeding costs and labor costs; the price will continue to increase, which brings a high-cost problem at the same time. But considering the highly physiological, anatomical, and histological similarities of NHPs to humans, therefore, NHPs are worth using.

### 3.4. Ethics and Morality of Animal Models

Animal experimental ethics is a problem that must be faced by scientific research. The main problem in China is the contradiction between animal protectionism and experimental animal science, as well as the differences in culture and concepts in different regions. In recent years, the trend of keeping pets has developed rapidly, which makes people have strong feelings for animals. Without knowing the background of scientific research, they have some prejudices against animal experiments. At the same time, the laws and regulations in this field are not perfect, which leads to some bad phenomena. However, some principles have been introduced internationally to help solve such problems, such as the implementation of the 3Rs principle. 3Rs include the use of unconscious experimental materials to replace live animals (replacement), reduce the number of experimental animals in the experiment (reduction), and improve animal welfare (refinement) in the whole process of the experiment [84–89]. At present, this principle has also become a recognized global animal scientific standard in animal ethics. Therefore, the use of NHPs for research must be subject to strict moral review before it can be approved. For example, is it scientifically necessary to use NHPs? Is the design scientific? Is the degree of injury appropriate? These moral reviews will also promote the healthy development of animal ethics. In view of the challenges and difficulties faced by NHP experiments, some researchers have also put forward another point of view of animal experiments, that is, use high-throughput approaches to overcome these problems. Although this view is highly feasible, there is still a long way to go before it can be fully achieved. But on special scientific issues, if animal models must be used in experiments, they should also be used after strict scrutiny and ethical considerations.

## 4. Conclusion and Prospect

An important method for studying mechanisms of human disease and developing disease treatments is to establish effective animal models. However, many rodent disease models, such as PD, RTT, DMD, and ADS, are not able to recapitulate the same process in humans, leading to difficulties in using rodents to study these diseases. NHPs are very close to humans in terms of evolution, development, metabolism, and pathology. Fortunately, NHP models of these diseases show similar symptoms to human patients. Therefore, NHPs are the ideal animal models for studying human diseases, especially complex diseases.

Embryo engineering combined with different gene editing methods, in theory, can construct various NHP disease models. There are different methods to establish an animal model at different stages of embryo formation, such as sperm vector, SCNT, pronuclear microinjection, retrovirus infection, and embryonic stem cell method. SCNT has been successfully applied in NHPs, providing an efficient and practical way to construct NHP disease models. Because different gene editing methods and stages have different effects on the mutation rate, embryonic development conditions, and birth rate of NHPs, those methods should be selected according to different needs.

The most popular gene editing tools include virus-mediated transgene, ZFNs [90], TALENs [33, 91, 92], and the CRISPR/Cas system [93, 94]. Since the CRISPR/Cas9 system has the advantages of relatively simpler operation, shorter experimental period, and being more versatile and efficient than other gene editing methods, most NHP disease models were constructed by it. However, it is also necessary to improve the repair efficiency of HDR or develop new methods to achieve the true “precision gene editing.” Nowadays, many new gene editing methods are emerging. For example, homology-independent targeted integration (HITI) strategy, a gene editing method based on NHEJ repair, can achieve targeted knockin in both mitotic and postmitotic cells [95]. The prime editing system includes three elements: Cas9 nickase, reverse transcriptase, and pegRNA. Interestingly, pegRNA has the dual role of guiding the other two elements to the target gene site and serving as a template for reverse transcription. In this way, prime editing could achieve targeted gene insertion, deletion, and all 12 types of point mutation without the donor template and DSBs [96]. The DddA-derived cytosine base editor (DdCBE) could achieve cytosine deamination modification on mtRNA without unwinding the dsDNA [97]. With the emergence of these new technologies, now we can generate more animal models, bringing new hopes to the research and treatment of related diseases.

Due to the advancement of the gene editing technology, *in vivo* gene therapy becomes possible. How to deliver gene editing systems to target cells or organs has become an urgent problem. The primary delivery method *in vitro* is nonviral delivery systems (electroporation, microinjection, and lipid nanoparticles), and the *in vivo* delivery method is viral delivery systems (AAV and lentivirus) [98]. With the widespread use of viral vectors, potential problems are also gradually revealed. At present, the main problem faced by therapeutic genome editing is the safety and effectiveness of the delivery system [99]. In NHPs, a viral delivery system (AAV) was used to deliver meganuclease [100] and nonviral vectors (lipid nanoparticles) to deliver ABE [101]; they specifically reduce the expression of *PCSK9*. The results showed that LDL-C levels were significantly reduced for a long time, providing a safe and effective strategy for the treatment of cardiovascular diseases. However, intravenous injection of AAV in large doses can cause acute liver failure and shock in monkeys [102], suggesting that the use of AAV needs to consider more factors and safer delivery methods need to be developed. In general, the use of NHP models can more comprehensively reflect the safety and effectiveness of delivery systems or gene editing strategies, providing a strong reference for clinical applications and transformation.

In summary, the construction of NHP disease models provides a valuable research platform for human diseases, especially for those complicated diseases that cannot be successfully modeled in other animals. The use of NHP models to verify disease treatment strategies before the clinic can predict treatment effects more accurately and provide a powerful reference for clinical treatment. NHP disease models have promoted the development of translational medicine and brought new hopes to understand the underlying disease mechanisms and explore disease treatment methods. Therefore, NHPs have an indispensable status in the field of life sciences.

## Figures and Tables

**Table 1:** NHPs models constructed by different methods.

Type of models	Year	Species	Method	Gene	Stages	Samples	Implanted blastocysts	Pregnancy rate^#^	Total neonatus	Models	Mutation rate^#^	Contribution	Author/reference
Viral vector-mediated transgenic	2001	Rhesus monkeys	Retroviral vector	*GFP*	Mature oocytes	224 oocytes	40	5/20 = 25%	3	1	33%	The first transgenic monkey	Chan et al. [17]
	2008	Rhesus monkeys	Lentivirus vector	*HTT-84Q* and *GFP*	Mature oocytes	130 oocytes	30	6/8 = 75%	5	5	100%	The first monkey model of HD	Yang et al. [23]
	2009	Common marmosets	Lentiviral vector	*EGFP*	Preimplantation embryos	201 embryos	91	7/50 = 14%	5	5	100%	The first germline transmission	Sasaki et al. [18]
	2010	Rhesus monkeys	Lentiviral vector	*EGFP*	Early cleavage-stage embryos	70 embryos	30	5/8 = 63%	5	2	40%	The first transgenic monkey in China	Niu et al. [26]
	2015	Rhesus monkeys	Lentiviral vector	*α-Syn* (*A53T*)	Mature oocytes	133 oocytes	75	11/25 = 44%	7	6	86%	The first monkey model of PD	Niu et al. [27]
	2016	Cynomolgus monkeys	Lentiviral vector	*MECP2*	Mature oocytes	First: 94 oocytes; second: 264 oocytes	53 105	9/18 = 50%; 7/36 = 19%	8; 2	Total 8	80%	The monkey model of autism-like disease	Liu et al. [28]
	2016	Cynomolgus monkeys	Lentiviral vector	*GFP*	24 hours after ICSI; 4 hours before ICSI	18; 69	5; 17	2/3 = 67%; 5/17 = 29%	0; 2	0; 2	0%; 100%	Construction of cynomolgus monkeys expressing GFP	Seita et al. [25]
	2019	Rhesus monkeys	Lentivirus vector	*MCPH1*	Early cleavage-stage embryos	—	—	—	6	6	100%	The huMCPH1 transgenic monkey	Shi et al. [30]

TALEN or ZFN knockout	2014	Rhesus monkeys and cynomolgus monkeys	TALEN	*MECP2*	One-cell embryos	Rhesus: 59 embryos cynomolgus: 86 embryos	21; 54	2/7 = 29%; 6/19 = 32%	0; 1	Total 4	—	The first monkey model of RTT; the first monkey model of TALEN	Liu et al. [33]
	2017	Cynomolgus monkeys	TALEN	*MECP2*	One-cell embryos	123 embryos	123	14/41 = 34%	7	6	86%	Research on RTT	Chen et al. [34]
	2016	Cynomolgus monkeys	TALEN	*MCPH1*	Embryos	55 embryos	52	3/9 = 33%	3	1	33%	The first monkey model of human microcephaly	Ke et al. [19]
	2016	Common marmosets	ZFN and TALEN	*IL2RG*	Pronuclear stage embryos	250 embryos	179	19/113 = 17%	21	9	43%	The first monkey model of ZFN; the first monkey model of X-SCID	Sato et al. [35]

CRISPR/Cas9 knockout	2014	Cynomolgus monkeys	CRISPR/Cas9	*Pparg* and *Rag1*	One-cell embryos	186 zygotes	83	10/29 = 34%	5	2	40%	The first monkey model of CRISPR/Cas9	Niu et al. [36, 37]
	2015	Cynomolgus monkeys	CRISPR/Cas9	*P53*	Zygotes	108 zygotes	62	4/13 = 31%	3	2	67%	The first live p53 biallelic mutant monkey	Wan et al. [20]
	2015	Rhesus monkeys	CRISPR/Cas9	*Dystrophin*	Zygotes	488 embryos	179	17/59 = 29%	14	9	64%	The first monkey model of DMD	Chen et al. [38]
	2015	Cynomolgus monkeys	CRISPR/Cas9	*Nr0b1* (*Dax1*)	One-cell embryos	186 zygotes	83	10/29 = 34%	5	—	—	The first monkey model of AHC-HH	Kang et al. [39]
	2017	Cynomolgus monkeys	CRISPR/Cas9	*SHANK3*	One-cell embryos	116 embryos	116	3/37 = 8%	1	1	100%	The first monkey model of ASD	Zhao et al. [45]
	2018	Cynomolgus monkeys	CRISPR/Cas9	*SIRT6*	Zygotes	98 zygotes	48	4/12 = 33%	3	3	100%	The first monkey model of perinatal lethality syndrome	Zhang et al. [46]
	2019	Cynomolgus monkeys	CRISPR/Cas9	*SHANK3*	Embryos	178 embryos	178	12/26 = 46%	9	5	56%	Monkey model of ASD and Phelan–McDermid syndrome	Zhou et al. [47]
	2019	Cynomolgus monkeys	CRISPR/Cas9	*BMAL1*	Zygotes	88 embryos	88	10/31 = 32%	8	5	63%	The first monkey model of circadian and psychiatric disorders	Qiu et al. [48]
	2019	Cynomolgus monkeys	CRISPR/Cas9	*PKD1*	Embryos	423 embryos	86	29/86 = 34%	14	19	—	The first monkey model of ADPKD	Tsukiyama et al. [49]
	2019	Rhesus monkeys	CRISPR/Cas9	*PINK1*	One-cell embryos	158 embryos	87	11/28 = 39%	11	8	73%	Monkey model of PD	Yang et al. [50]
	2019	Cynomolgus monkeys	CRISPR/Cas9	*HBB*	Zygotes	97 zygotes	22	—	1	1	100%	The first monkey model of human *β*-thalassemia	Huang et al. [51]
	2019	Rhesus monkeys	CRISPR/Cas9	*SHANK3*	Pronuclear-stage embryos	mRNA: 22; nuclease: 26	Editing efficiency: mRNA:80%; nuclease:100%					KI/KO efficiency verified at embryo level	Kumita et al. [54]
				*c-kit*	Pronuclear stage embryos	mRNA: 20; nuclease: 25	Editing efficiency: mRNA:77.8%; nuclease:100%						

CRISPR/Cas9 knockin	2017	Cynomolgus monkeys	CRISPR/Cas9	*mCherry*	Zygotes	First: 26; second: 10	First (high-concentration HMEJ donor): 4/5 mCherry^+^ blastocysts; second (low-concentration HMEJ donor): 1/4 mCherry^+^ blastocysts					Verified HMEJ-based knockin at the embryo level	Yao et al. [55]
	2018	Cynomolgus monkeys	CRISPR/Cas9	*Oct4-GFP*	Zygotes	198 zygotes	120	12/40 = 30%	8	1	13%	The first monkey model of KI	Cui et al. [56]
	2019	Rhesus monkeys	CRISPR/Cas9	*c-kit*	Pronuclear stage embryos	40 embryos	Optimal injection combination: 36 nt sense ssODN and CRISPR/nuclease mixture; editing efficiency: 30.8%					KI/KO efficiency verified at embryo level	Kumita et al. [54]

Chimera embryo	2015	Cynomolgus monkeys	Retroviral vector	*GFP*	ESC	—	14	1/5 = 20%	0	2	—	The first monkey chimeras	Chen et al. [22]

SCNT	2018	Cynomolgus monkeys	—	—	—	—	—	6/21 = 29%	2	2	100%	The first monkey model of SCNT	Liu et al. [16]
	2019	Cynomolgus monkeys	—	—	—	—	325	16/65 = 25%	5	5	100%	The first disease monkey model of SCNT	Liu et al. [64]

BE point mutation	2020	Cynomolgus monkeys	CBE	*LMNA*	Zygotes	86 zygotes	41	6/11 = 55%	5	4	80%	The first monkey model of BE	Wang et al. [60]
	2020	Rhesus monkeys	CBE	*MECP2*	Zygotes	—	Embryo level					Established a stable embryo editing system of RTT	Zhou et al. [62]
	2020	Cynomolgus monkeys	CBE and ABE	Multiple loci	Zygotes	—	Embryo level					Simultaneously edit multiple loci at the embryo level	Zhang et al. [63]

Pregnancy rate^#^: pregnancy/surrogate^∗^100%; mutation rate^#^: mutation neonatus/total number of neonates^∗^100%.
